# Evaluation of ^99m^Tc-HYNIC-MPG as a novel SPECT radiotracer to detect EGFR-activating mutations in NSCLC

**DOI:** 10.18632/oncotarget.17251

**Published:** 2017-04-19

**Authors:** Zunyu Xiao, Yan Song, Wang Kai, Xilin Sun, Baozhong Shen

**Affiliations:** ^1^ TOF-PET/CT/MR Center, The Fourth Hospital of Harbin Medical University, Harbin, China; ^2^ Molecular Imaging Research Center, Harbin Medical University, Harbin, China

**Keywords:** ^99m^Tc, HYNIC-MPG, SPECT imaging, mutant EGFR, non small cell lung cancer (NSCLC)

## Abstract

Tyrosine kinase inhibitors (EGFR-TKIs) targeting the epidermal growth factor receptor (EGFR) have been used in non-small cell lung carcinoma (NSCLC) for years with promising results, in particular in patients with activating mutations in the EGFR kinase domain (exon 19 E746-A750 deletion or exon 21 L858R point mutation). However, despite their great success in the clinic, a significant number of patients do not respond to EGFR-TKIs, such as those carrying the L858R/T790M mutation or EGFR wild type. Thus, detecting the EGFR mutation status before EGFR-TKIs therapy is essential to ensure its efficacy. In this study, we report a novel SPECT tracer ^99m^Tc-HYNIC-MPG that binds specifically to activating mutant EGFR and which could therefore be used to noninvasively select patients sensitive to EGFR-TKIs. We evaluated the capacity of ^99m^Tc-HYNIC-MPG in detecting EGFR-activating mutations both *in vitro* and *in vivo* using four human NSCLC cell lines (PC9, H1975, H358 and H520). ^99m^Tc-HYNIC-MPG had significantly higher accumulation in PC9 tumor cells when compared to H1975, H358 and H520 tumors cells, which may be due to the activating mutations (exon 19 deletion) in EGFR tyrosine kinase domain in PC9 cells. Thus, ^99m^Tc-HYNIC-MPG SPECT imaging may be used to identify NSCLC tumors with a potential high response rate to EGFR-TKIs.

## INTRODUCTION

Lung cancer has the highest morbidity rates of cancer disease, in particular non-small cell lung carcinoma (NSCLC), which accounts for about 80% of lung cancers [1]. While surgery, radiotherapy and chemotherapy have been used to treat NSCLC for several years, the five year survival rate unfortunately remains less than 2% [2]. Tyrosine kinase inhibitors targeting the epidermal growth factor receptor (EGFR-TKIs) have been recently developed and appear to work effectively against several types of cancer, including NSCLC [3]. EGFR is a member of the HER family of transmembrane tyrosine kinase receptors that leads to the production of the proto-oncogene c-erbB1. EGFR plays one of the fundamental roles in the regulation of numerous cellular and physiological processes in homeostasis and during tumorinogenesis, such as cell growth, proliferation, differentiation, angiogenesis and metastasis. EGFR has three domains: an extracellular ligand-binding region, a single transmembrane helix and a cytoplasmic segment [4]. EGF and TGFa(transforming growth factor alpha) ligands, as well as other antigens, bind to the extracellular domain thereby activating the downstream signaling pathways [5]. In NSCLC, an EGFR mutation in the intracellular region has been often identified in correlation with sensitivity to EGFR-TKIs [6]. However, while first generation EGFR-TKIs gefitinib (Iressa, ZD1839; AstraZeneca) and erlotinib (Tarceva, OSI-774; Genentech) have worked well in a subset of patients carrying mutations in the EGFR kinase domain in NSCLC [7], a significant proportion developed primary and secondary drug resistance during treatment [8]. About half of the drug sensitive NSCLC patients harbor a secondary mutation in the EGFR [9] that prevents effective inhibition by EGFR-TKIs due to steric hindrance or increased binding affinity for adenosine triphosphate(ATP) [10]. Thus, detecting the EGFR-activating mutation status prior to treatment is the key for predicting the efficacy of EGFR-TKI therapy in NSCLC patients.

Direct gene sequencing is the standard method used for detection of EGFR mutations in cancer, however, its many limitations, such as low sensitivity and length of procedure (several days), restrict its use in the clinic. Furthermore, gene sequencing cannot reflect EGFR mutation status in the whole tumor. Thus, a noninvasive method to identify patients sensitive to EGFR-targeted therapy and to monitor the EGFR mutation status in real-time during treatment with EGFR-TKIs is urgently needed. Since 1999, when Ralph weissleder from Harvard University first reported the noninvasive imaging technology–Molecular Imaging, it is a useful tool that can measure and characterize a variety of biological processes at the cellular or molecular levels *in vivo* [11], and which could therefore be utilized to identify EGFR activating mutations and to assess tumor response to EGFR-TKIs therapy *in vivo*. Notably, some PET(Positron emission tomography) tracers, such as ^18^F-PEG6-IPQA [12, 13], ^11^C-erlotinib, ^11^C-PD153035 and ^11^C-gefitinib [14–16], and some antibodies [17], have been synthesized and used to evaluate the mutation status of EGFR in NSCLC.

PD153035 is a EGFR tyrosine kinase inhibitor that could bind to activating mutant EGFR(19 exon deletion and L858R point mutation) and wild-type EGFR, but it can not binds to L858R/T790M mutant EGFR, and the capacity of PD153035 binds to activating mutant EGFR is much higher than that in wild-type EGFR. We previously reported a novel SPECT(Single-Photon Emission Computed Tomography) probe, HYNIC-MPG (2-(2-(2-(2-(4-(3-chloro-4- fluorophenylamino)-6-methoxyquinazolin-7-yloxy)ethoxy)ethoxy)ethoxy)ethyl-6-hydrazinylnicotinate hydrochloride), a analogue of PD153035, which could be labeled by ^99m^Tc for targeting mutant (activity) EGFR [18], and we evaluated its chemical and biological properties, including its EGFR binding capacity *in vitro* and *in vivo*. In the current study, we further explore the potential of ^99m^Tc-HYNIC-MPG as a novel SPECT radiotracer for the *in vivo* identification of EGFR exon 19 deletion in NSCLC xenografts.

## RESULTS

### Cell uptake, efflux and blocking assay of ^99m^Tc-HYNIC-MPG

**Figure 1:**
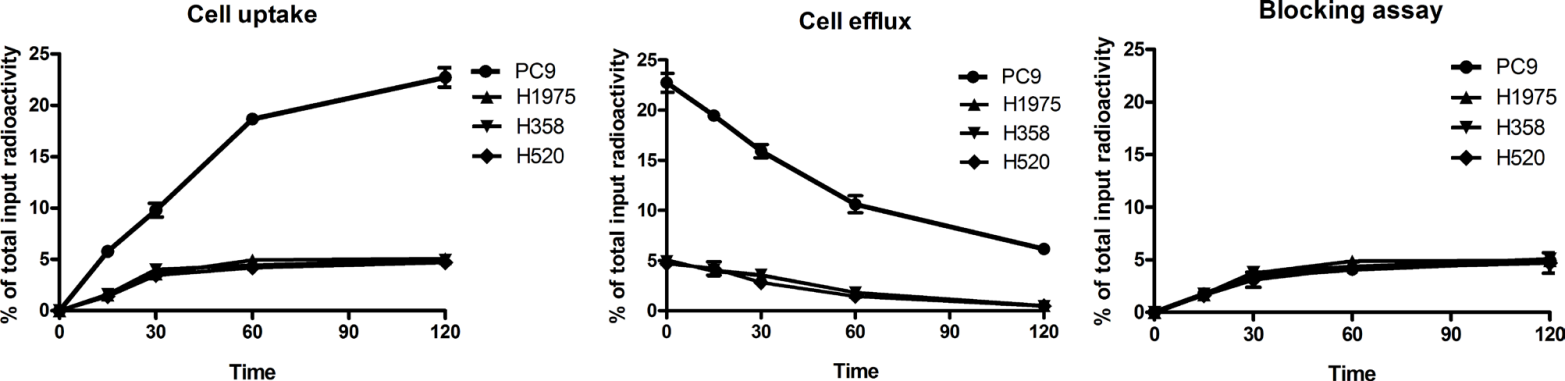
Cell uptake, efflux and blocking assay of ^99m^Tc-HYNIC-MPG in NSCLC cell lines

Cellular uptake and efflux experiments were performed to validate the specificity of ^99m^Tc-HYNIC-MPG in cell levels (Figure 1). PC9 cell lines have a higher uptake ratio than that of H1975, H358 and H520 in all time points examined. At 2h, the accumulation of ^99m^Tc-HYNIC-MPG in PC9 cells reached the highest level at 22.73 ± 1.63% of total input activity, which is about 4.4, 4.6 and 4.8 times higher than H1975, H358 and H520, respectively (*P* < 0.01). However, the high uptake of ^99m^Tc-HYNIC-MPG in PC9 cells was blocked in the presence of 100 uM PD153035, and the uptake ratio decreased from 22.73 ± 1.63% to 4.89 ± 0.08% of total input activity (*P* < 0.01), and there is no significant change in the other three NSCLC cell lines. This result suggests that the high uptake rate of ^99m^Tc-HYNIC-MPG in PC9 cells is related to the expression levels of EGFR exon 19 E746-A750 deletion mutant proteins. In the cell efflux assay, ^99m^Tc-HYNIC-MPG was equally well retained in PC9 cells, even at 2h, and the ratio of total input activity in PC9 cells remained at 6.16 ± 0.28%, which was much higher than that of H1975, H358 and H520 cells. These data indicate that ^99m^Tc-HYNIC-MPG might be used for long-term monitoring of the dynamic process of EGFR mutation status at the cellular level.

### *SPECT* imaging and blocking assay

**Figure 2:**
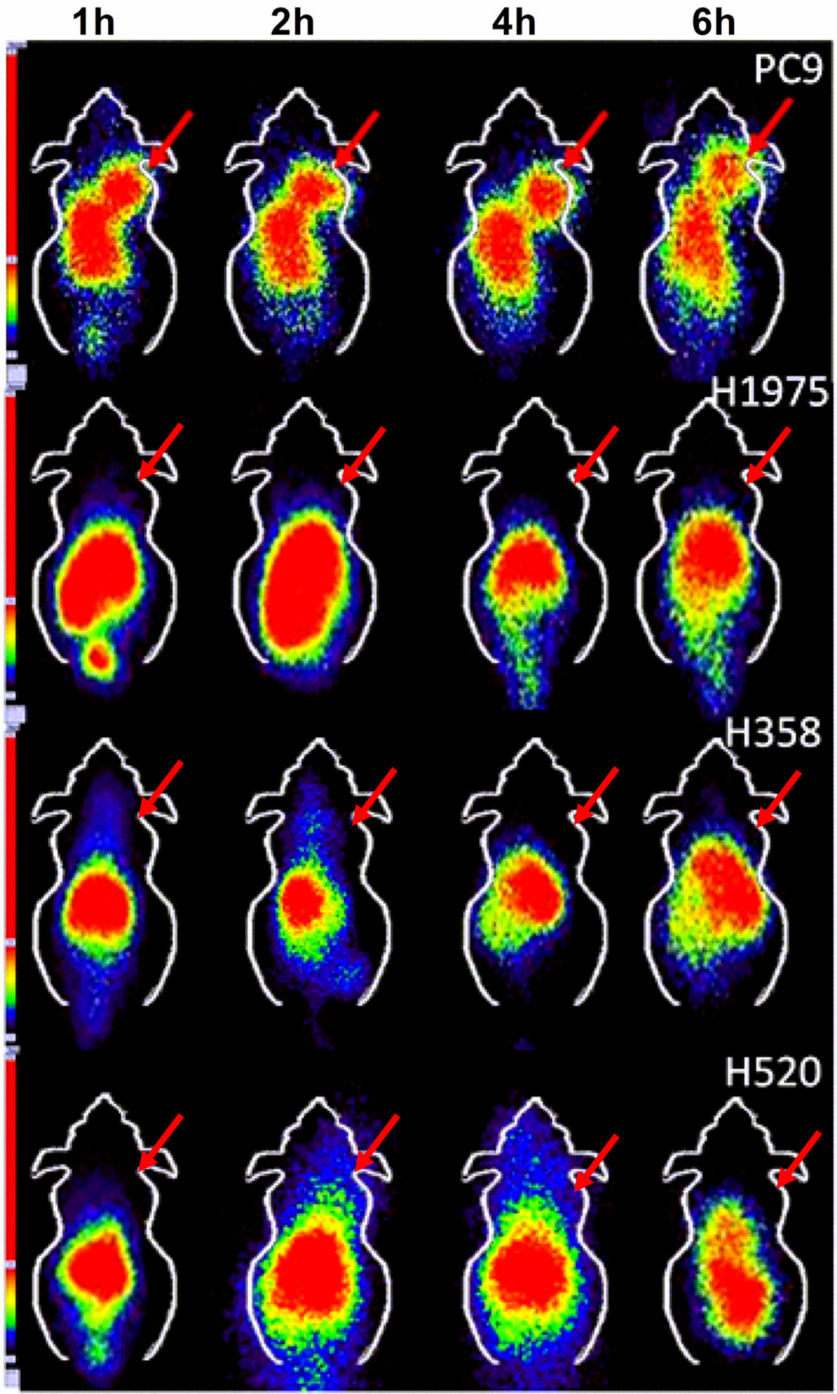
SPECT imaging of ^99m^Tc-HYNIC-MPG in PC9, H1975, H358 and H520 xenografts at 1 h, 2 h, 4 h, and 6 h time points

**Figure 3:**
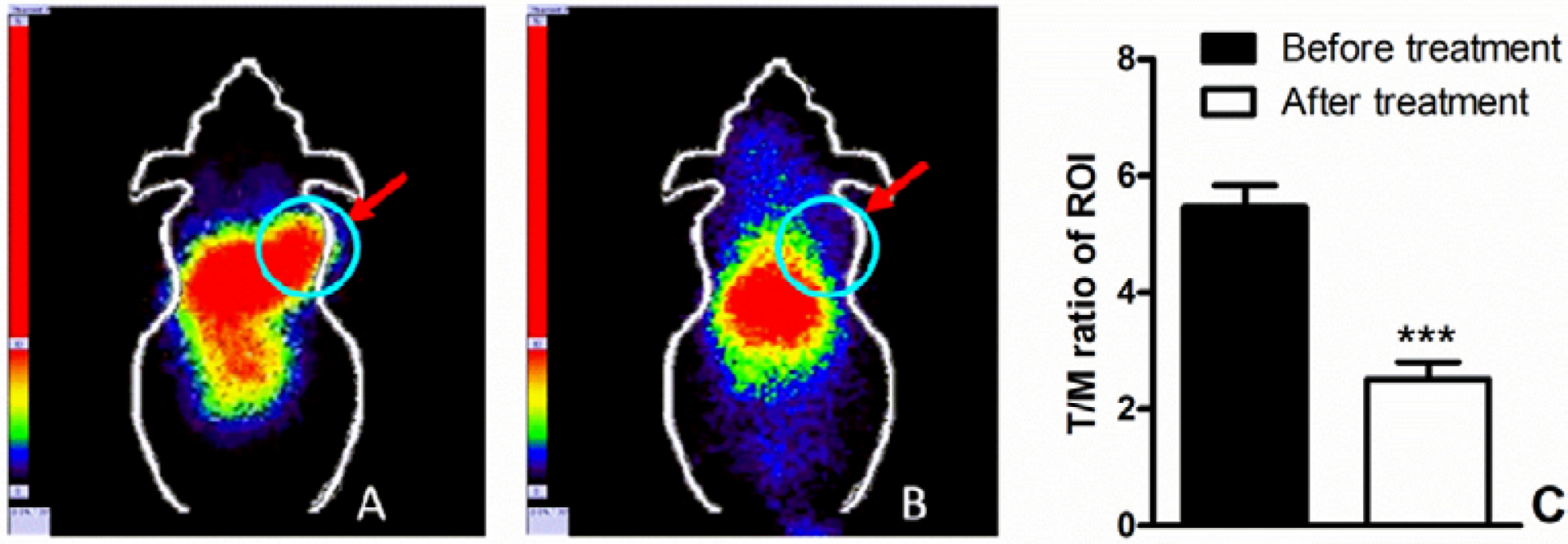
(**A**) 200 uci of 99mTc-HYNIC-MPG was injected via the tail vein, 2 h later, SPECT imaging was performed on PC9 xenografts; (**B**) 100 mg/Kg PD153035 was injected before SPECT imaging; T/M ratio before and after treatment was shown in (**C**).

*In vivo* SPECT scans were performed in four xenograft groups (PC9, H1975, H358, H520); each group had three tumor-bearing mice. The uptake of ^99m^Tc-HYNIC-MPG in the PC9 tumor was significantly higher than in the other tumors at all time points examined (Figure 2). Tumor to muscle (T/M) ratios were calculated over the region of interest (Table 1). 2 h after the injection of ^99m^Tc-HYNIC-MPG into the tail vein, the T/M ratio in the PC9 group reached 5.47 ± 0.37, versus that of 2.45 ± 0.32 in the H1975 group, 2.35 ± 0.40 in the H358 group and 1.72 ± 0.28 in the H520 group (*P* < 0.01). The accumulation of ^99m^Tc-HYNIC-MPG in the PC9 tumor was significantly decreased at the presence of 100 mg/Kg PD153035 and the T/M ratio was also decreased to 2.51 ± 0.30 (Figure 3), suggesting that high uptake of ^99m^Tc-HYNIC-MPG in the PC9 tumor is related to the EGFR-activating mutation.

**Table 1:** Tumor to muscle ratio of ROI

	1 h	2 h	4 h	6 h
**PC9**	3.63 ± 0.23	5.47 ± 0.37	4.21 ± 0.35	4.11 ± 0.36
**H1975**	1.67 ± 0.09	2.45 ± 0.32	1.81 ± 0.08	1.62 ± 0.21
**H358**	1.55 ± 0.20	2.35 ± 0.40	1.66 ± 0.40	1.32 ± 0.23
**H520**	1.37 ± 0.18	1.72 ± 0.28	1.64 ± 0.24	1.19 ± 0.19

Data was analyzed by mean ± SD (*n* = 3).

### *Ex vivo* biodistribution

**Figure 4:**
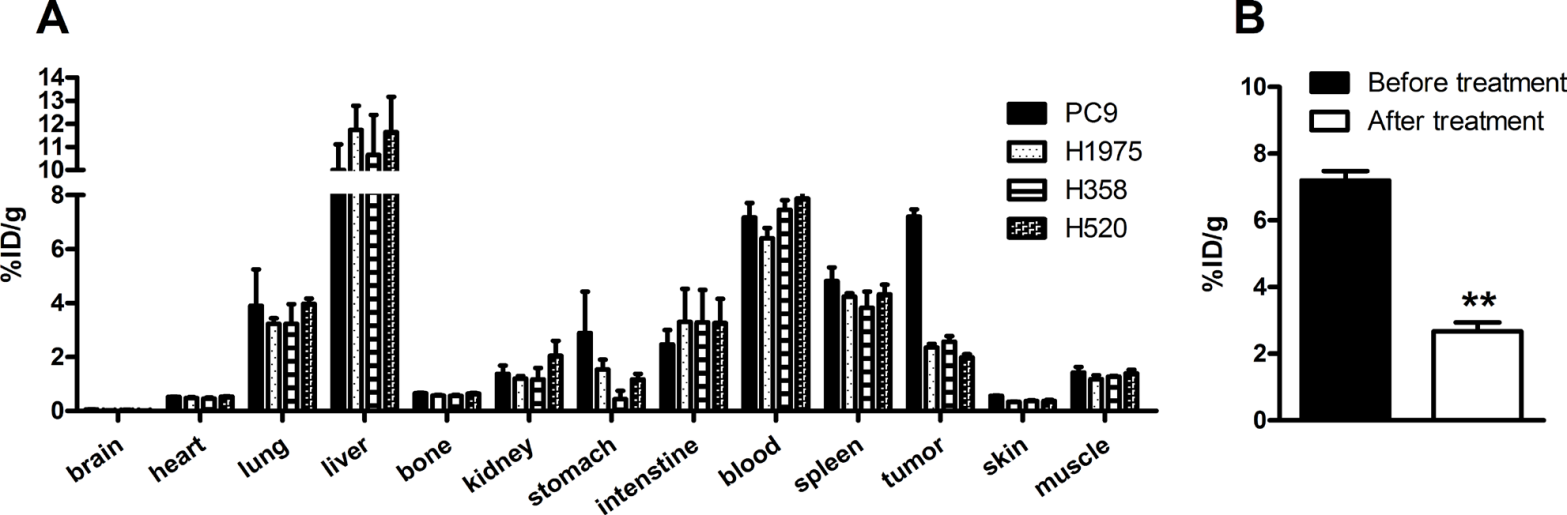
(**A**) biodistribution of 99mTc-HYNIC-MPG in major organs at 2 h time point; (**B**) The uptake of 99mTc-HYNIC-MPG in PC9 tumor reached 7.20 ± 0.27% ID/g 2 h post injection through the tail vein, and was blocked at the presence of 100 mg/Kg PD153035.

To quantify the SPECT imaging results of ^99m^Tc-HYNIC-MPG in xenografts, *ex vivo* biodistribution was monitored at 1 h, 2 h, 4 h and 6 h (Supplementary Tables 1–4). The uptake of ^99m^Tc-HYNIC-MPG in the PC9 tumor reached 7.20 ± 0.27% ID/g at 2h post injection, compared to 2.35 ± 0.14% ID/g in H1975, 2.57 ± 0.20% ID/g in H358 and 1.98 ± 0.13% ID/g in H520 tumors (*P* < 0.01), shown in Figure 4. The T/M ratio of ^99m^Tc-HYNIC-MPG accumulation in the PC9 group at 2h was 5.14 ± 0.83. In contrast, the T/M ratio was only 2.01 ± 0.19 in H1975, 2.02 ± 0.19 in H358 and 1.42 ± 0.04 in H520 tumors. In the presence of 100 mg/Kg PD153035, the uptake of ^99m^Tc-HYNIC-MPG in PC9 tumors decreased from 7.20 ± 0.27% ID/g to 2.67 ± 0.27% ID/g, and the T/M ratio of ^99m^Tc-HYNIC-MPG accumulation in the PC9 group was reduced to 2.32 ± 0.18. Thus, this *ex vivo* biodistribution data is in accord with the SPECT imaging results. As expected, the highest accumulation of ^99m^Tc-HYNIC-MPG occurred in the liver, as this is the largest metabolic organ and the pharmacokinetics of these small molecules takes places mainly in the liver. However, adding 4 ethylene glycol to PD153035 decreased the ^99m^Tc-HYNIC-MPG uptake ratio in the liver (16.89 ± 1.56), in agreement with what was previously described [18]. As PEGs(polyethylene glycol) may reduce the liposolubility of the probe, these results suggest that PEGs facilitate the access of ^99m^Tc-HYNIC-MPG to the tumor region by preventing its accumulation in the liver. The uptake of ^99m^Tc-HYNIC-MPG in the brain was very low in all tumor-bearing mice at all time points examined, suggesting that ^99m^Tc-HYNIC-MPG cannot cross the blood-brain barrier.

### Western blot analysis and immunofluorescence Staining

**Figure 5:**
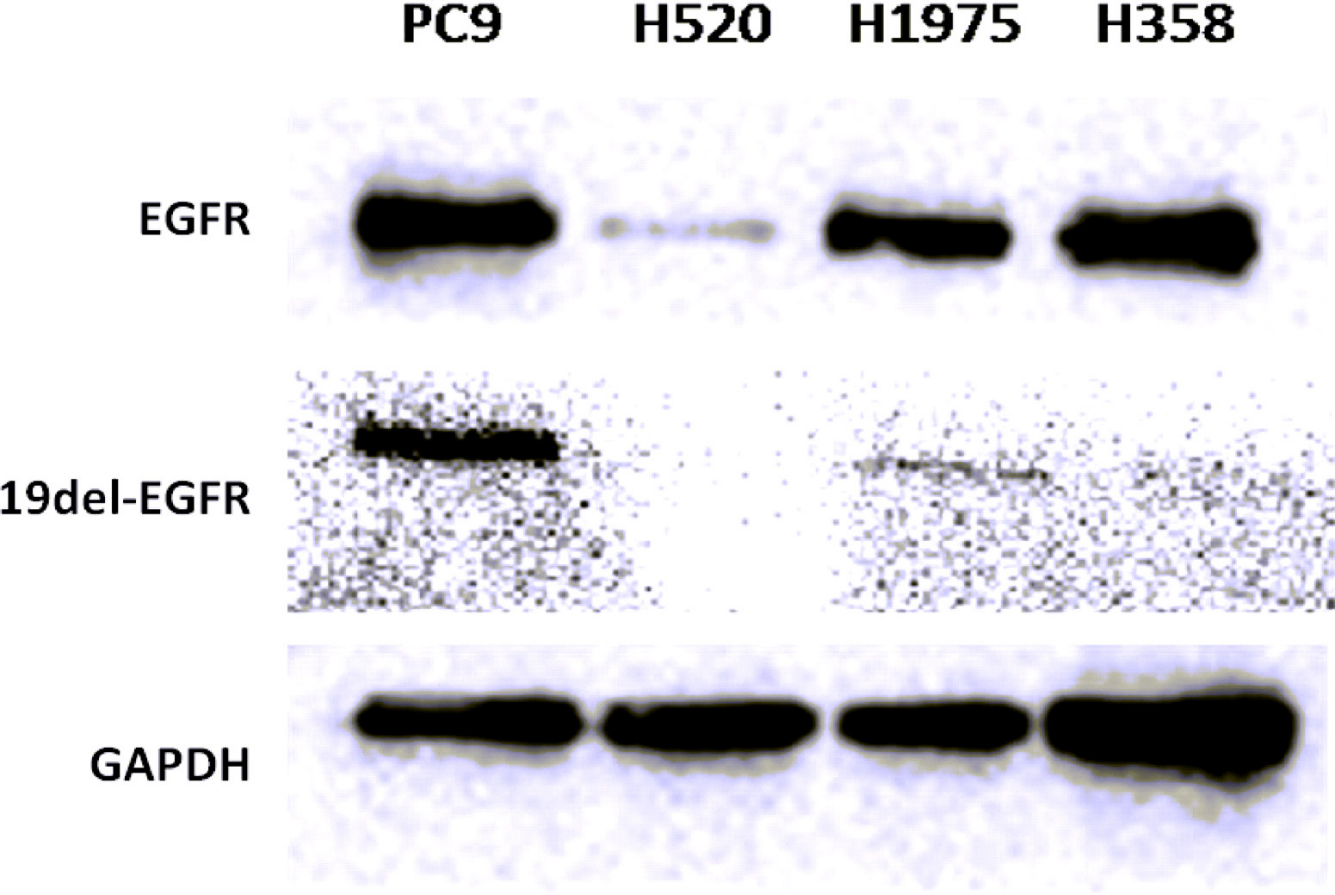
Western blot of EGFR and 19del-EGFR expression level in 4 NSCLC tumors

**Figure 6:**
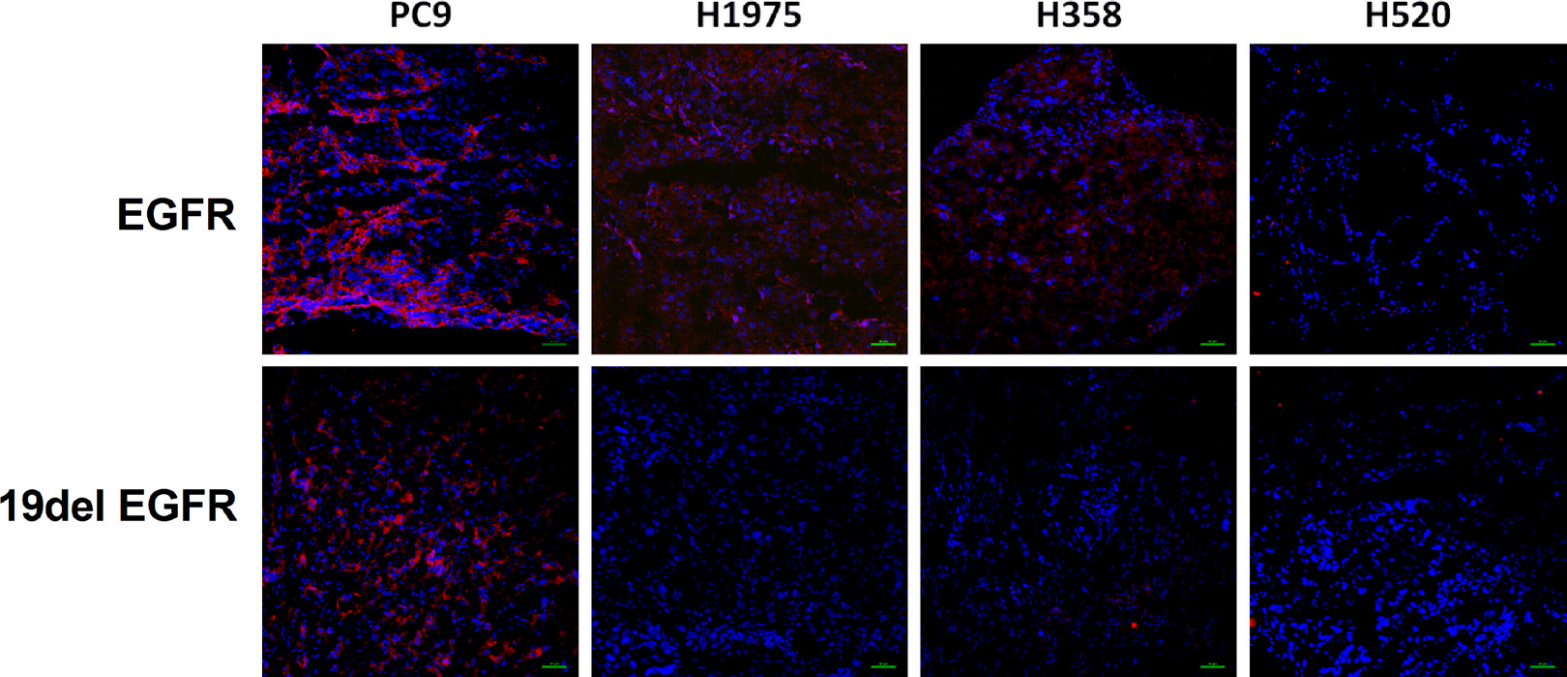
Immunofluorescence staining of EGFR and EGFR exon 19 (19del EGFR) expression level in tumor tissues Above, red color represents EGFR expression, while blue color represents cell nuclei; blow, red color represents the level of EGFR exon 19 deletion, while blue color represents cell nuclei.

Expression levels of wild type EGFR and EGFR exon 19 E746-A750 deletion proteins were quantified in PC9, H1975, H358 and H520 NSCLC tumors by western blot assays and immunofluorescence staining (Figure 5 and Figure 6). PC9, H1975 and H358 all express EGFR protein, but PC9 also expresses EGFR 19 deletion protein, suggesting that the high uptake of ^99m^Tc-HYNIC-MPG in PC9 is likely due to the presence of EGFR exon 19 E746-A750 deletion protein.

## DISCUSSION

Currently, the most widely used methods for detecting EGFR mutations in NSCLC are gene sequencing, fluorescent *in situ* hybridazation and immunohistochemical assays, however, they present important limitations for EGFR-TKIs therapy. First, analyses from aspiration biopsies cannot reflect the EGFR mutation status of a whole tumor due to its heterogeneity. Second, repeated aspiration biopsies in advanced NSCLC can cause psychological and physical harm, which most of NSCLC patients cannot tolerate. In contrast, noninvasive SPECT imaging offers enormous potential to overcome these hurdles. Indeed, we have shown here that a ^99m^Tc-HYNIC-MPG radiotracer can selectively accumulate in NSCLC as a result of EGFR expressing levels and mutation status in the tumor region. These results demonstrate that noninvasive SPECT imaging could be used to select patients sensitive to EGFR-TKIs therapy.

EGFR mutations are an excellent target for TKIs and occur in 10–35%of NSCLCs [19]. We have recently developed a novel SPECT tracer (^99m^Tc-HYNIC-MPG) that binds specifically and stably to EGFR 19 deletion proteins *in vitro*[18]. Previously others have utilized alternative radionuclide labeling with EGFR-TKIs, for instance, ^18^F-gefitinib, ^11^C-PD 153035 or ^11^C-gefitinib, among others [14–16]. However, the half-lives of ^18^F and ^11^C are 110 min and 20.34 min respectively, which is not sufficient for real-time monitoring of EGFR-TKIs efficacy *in vivo* [20–22]. In addition, the procedures for synthesizing these probes are highly complex, time-consuming and costly, which greatly limits their availability from commercial manufacturers. In contrast, ^99m^Tc can label many compounds for imaging in nearly every organ *in vivo* at low cost. Furthermore, ^99m^Tc has a long half-life (6.02 h), a good range of γ ray energy (140 keV) and it is easy to transport, which facilitates its clinical application [23, 24].

While currently available probes targeting EGFR bind to the extracellular region of both wild type and mutant proteins, ^99m^Tc-HYNIC-MPG binds specifically to EGFR 19 E746-A750 deletion proteins. This binding specificity offers the unique advantage of allowing the identification of patients carrying mutations sensitive to EGFR-TKI therapy. Furthermore, with its high sensitivity and stability, ^99m^Tc-HYNIC-MPG has the optimal properties for real-time monitoring of the EGFR mutation status during EGFR-TKI therapy *in vivo*. However, this promising probe has some limitations that should be addressed in future investigations. For instance, the detailed mechanism for ^99m^Tc-HYNIC-MPG targeting to mutant EGFR remains unclear. Furthermore, although nonspecific uptake of ^99m^Tc-HYNIC-MPG to some organs is reduced when compared to other probes, it is still not low enough for clinical use, and it should therefore be improved by direct modification or other methods. Finally, small animal imaging SPECT equipment could be used to improve the quality of image.

In conclusion, ^99m^Tc-HYNIC-MPG SPECT imaging is a promising approach for selecting NSCLC tumors carrying EGFR exon 19 deletion mutation sensitive to EGFR-TKIs therapy. In view of the capacity of binding to NSCLC EGFR 19 deletion protein, a clinical study is required.

## MATERIALS AND METHODS

### Radiosynthesis of ^99m^Tc-HYNIC-MPG

^99m^Tc-HYNIC-MPG was synthesized as previously described[18]: a mix of 0.5 ml EDDA solution (10 mg/ml), 10 μl HYNIC-MPG (3 mg/ml), 10 μl SnCl_2_ (0.2 mg/ml) and Tricine 20 mg with nitrogen protection was heated at 100°C for 10 min (all reagents were purchased from Sigma Company), then purified with C18 Sep-Pak plus cartridges. At the end of synthesis, the radiochemical purity of ^99m^Tc-HYNIC-MPG was > 98%.

### Cell culture

Four human NSCLC cell lines were used in this study: PC9 (EGFR 19 exon E746-A750 deletion), H1975 (EGFR L858R /T790M double mutation), H358 (EGFR wild type) and H520 (EGFR negative expression), which were all purchased from American Type Culture Collection (ATCC). Cells were grown in RPMI 1640 medium (HyClone) containing 10% fetal bovine serum (FBS) (HyClone) and then cultured at 37°C in a humidified atmosphere with 5% CO_2_.

### Cell uptake, efflux and blocking assays

Cell uptake: 5 × 10^6^ cells were seeded in 12-well plates 24 h prior to uptake assay. 37 KBq (1.0 μCi/ml) of ^99m^Tc-HYNIC-MPG was added to each well. At 15 min, 30 min, 1 h and 2 h time points, the media was removed and washed twice with 1 ml of ice-cold PBS. 200 μl 0.1 N NaOH was then added for dialysis of the cells. After dialysis the cells were washed twice with 200 μl of ice-cold PBS. The cells were recovered in PBS and counted with CPM (counts per minute) in a gamma counter (PerkinElmer 2480). For the blocking assay, PD153035 (100 μM) was added before ^99m^Tc-HYNIC-MPG in NSCLC cells. 1h later, the cell uptake was performed as mentioned above.

Cell efflux: Efflux assays were performed after 2 h incubation with ^99m^Tc-HYNIC-MPG. The media was then removed and replaced with serum free medium without ^99m^Tc-HYNIC-MPG. 15 min, 30 min, 1 h or 2 h later, the media was removed and the cells were washed twice with ice-cold PBS. 200 μl 0.1 N NaOH was added for dialysis of cells. The well was then washed twice with 200 μl ice-cold PBS. The cells were recovered in PBS and counted with CPM (counts per minute) in a gamma counter (PerkinElmer 2480).

### Animal xenograft models

Female BALB/c nude mice (mean weight ± SD, 20 ± 0.5 g; age, 6 week [Department of Animal Center, Shanghai]) were used to establish subcutaneous transplanted human NSCLC xenografts (PC9, H1975, H358, H520) for SPECT imaging and biodistribution. After anesthetization with 2% isoflurane in O_2_, 5 × 10^6^ cells were injected in the right shoulder. The tumor volume was observed every other day until it reached 250 mm^3^. The mice were maintained on a standard diet, bedding, and specific pathogen free environments. All animal experiments were approved by the Harbin Medical University Animal Ethics Committee in accordance with Chinese legislation and were performed following their guidelines. The institutional review board approved this study in mice with human tumor xenografts.

### SPECT imaging

SPECT scans and image analyses were done using a clinical SPECT scanner (Siemens e.cam Gamma Camera). Each PC9, H1975, H358 and H520 tumor-bearing mouse was injected in a tail vein with 7.4 MBq (200 μCi in 100 uL) of ^99m^Tc-HYNIC-MPG under isoflurane (2% in O_2_) anesthesia (*n* = 3 per group). SPECT scan was then performed at 1 h, 2 h, 4 h and 6 h time points. The mice were located in the center field view of the detector. The imaging procedure was completed with a static anterior image (5 min) and with a zoom factor of 1.00 and digitally stored at 1024 × 1024. The energy window setting was 126 keV to 154 keV and nuclide-^99m^Tc. For blocking studies, 100 mg/kg PD153035 was injected in PC9 xenografts 1h before SPECT imaging, and then SPECT scan was performed as described above. The ratio of tumor to muscle (T/M) was calculated over the region of interest.

### *Ex vivo* biodistribution

An average dose of 0.925 MBq (25 μCi) of ^99m^Tc-HYNIC-MPG was injected into xenografts of all NSCLC cell lines via the lateral tail vein. The mice were sacrificed at 1 h, 2 h, 4 h and 6 h time points. Brain, heart, lung, liver, tumor, bone, kidney, stomach, intestine, blood, spleen, skin and muscle were collected immediately. The samples were weighed in wet-weight and the radioactivity of each sample was measured by a gamma-counter (PerkinElmer 2480). After corrected decay of all measurements, the radioactivity was expressed as percentage of injected dose per gram (% ID/g).

### Western blot assay

After SPECT imaging, the xenografts were sacrificed immediately and the tumor tissues were harvested. The tumor tissues were extracted in modified RIPA buffer on cold ice and the concentration of each sample was measured with a BCA protein assay kit (Pierce Biotechnology, Inc). Total proteins were loaded and separated by SDS-PAGE (10%), transferred to a nitrocellulose membrane and then incubated with 5% non-fat milk blocking buffer at room temperature for 30min. The membrane was then incubated at 4°C overnight with primary antibodies including EGF Receptor(D38B1) Rabbit mAb antibody or EGF Receptor (E746-A750 Specific) Rabbit mAb antibody, which were purchased from Cell Signaling Technology and this was followed by incubation with anti-rabbit IgG HRP-linked antibody (Cell Signaling Technology) at room temperature for 1 hour. The bands were detected by an ECL western blotting detection system (BD).

### Immunofluorescence staining

Immunofluorescence staining was performed to determine the total EGFR and EGFR exon 19 E746-A750 deletion expression levels. Tumor tissues were frozen overnight at −80°C, then cut into 5 μm per slice, fixed with 4% cold paraformaldehyde (PFA) for 30 min and washed three times in PBS, then blocked with 10% normal goat serum for 1 h. The slices were then incubated with EGF Receptor (D38B1) Rabbit mAb antibody (1:50, Cell Signaling Technology) or EGF Receptor (E746-A750 Specific) Rabbit mAb antibody (1:100, Cell Signaling Technology) at 4°C overnight, followed by incubation with Goat anti-Rabbit IgG (H + L) or Alexa Fluor^®^ 594 conjugate (1:1000, ThermoFisher) secondary antibodies and washed five times with PBS buffer. The slices were mounted with 49-6-diamidino-2-phenylindole (DAPI)-containing mounting medium and observed in a confocal microscope (C2Si, Nikon).

### Statistical analysis

In our study, all data are expressed as mean ± SD. GraphPad Prism 5 software was used to make statistical analysis. Statistical differences between the groups were compared by using one-way ANOVA and *t* test. It was considered significant if *p* <0.05.

## SUPPLEMENTARY MATERIALS AND TABLES


