# Supplementary material for: Evaluation of 99mTc-HYNIC-MPG as a novel SPECT radiotracer to detect EGFR-activating mutations in NSCLC
Source: Oncotarget. 2017 Apr 19;8(25):40732–40. doi: 10.18632/oncotarget.17251 (PMC5522229; doi:10.18632/oncotarget.17251)
Supplement: Supplementary file 1 [file oncotarget-08-40732-s001.pdf]

Evaluation of ^{99m}Tc-HYNIC-MPG as a novel SPECT radiotracer to detect EGFR-activating mutations in NSCLC

Supplementary Materials

Supplementary Table 1: Biodistribution of ^{99m}Tc-HYNIC-MPG at 1 h time point

Organ	PC9	H1975	H358	H520
brain	0.03 ± 0.01	0.07 ± 0.02	0.08 ± 0.01	0.09 ± 0.01
heart	0.60 ± 0.09	0.65 ± 0.06	0.61 ± 0.03	0.65 ± 0.25
lung	3.40 ± 0.35	3.45 ± 0.79	3.22 ± 0.09	3.37 ± 0.08
liver	12.92 ± 1.53	16.18 ± 2.05	15.28 ± 1.19	16.89 ± 1.56
bone	0.65 ± 0.04	0.55 ± 0.03	0.53 ± 0.02	0.64 ± 0.03
kidney	1.45 ± 0.45	1.39 ± 0.32	2.31 ± 0.17	1.09 ± 0.67
stomach	0.35 ± 0.01	0.33 ± 0.03	0.31 ± 0.01	0.40 ± 0.05
intestine	0.26 ± 0.07	0.27 ± 0.03	0.27 ± 0.03	0.27 ± 0.06
blood	9.56 ± 1.39	7.96 ± 0.84	7.52 ± 0.47	8.75 ± 0.77
spleen	3.28 ± 0.12	3.41 ± 0.26	3.67 ± 0.50	3.61 ± 0.13
tumor	3.79 ± 0.65	1.96 ± 0.16	1.53 ± 0.20	1.48 ± 0.12
skin	0.51 ± 0.05	0.37 ± 0.04	0.36 ± 0.03	0.45 ± 0.04
muscle	1.15 ± 0.16	1.34 ± 0.11	1.34 ± 0.21	1.26 ± 0.20

Values are mean ± SD and expressed as %ID/g (n = 3).

Supplementary Table 2: Biodistribution of ^{99m}Tc-HYNIC-MPG at 2 h time point

Organ	PC9	H1975	H358	H520
brain	0.06 ± 0.01	0.02 ± 0.01	0.04 ± 0.01	0.02 ± 0.00
heart	0.52 ± 0.01	0.48 ± 0.03	0.46 ± 0.04	0.53 ± 0.01
lung	3.90 ± 1.35	3.23 ± 0.20	3.23 ± 0.73	3.97 ± 0.19
liver	10.01 ± 1.10	11.73 ± 1.05	10.66 ± 1.73	11.64 ± 1.52
bone	0.65 ± 0.02	0.57 ± 0.02	0.56 ± 0.03	0.64 ± 0.02
kidney	1.37 ± 0.31	1.20 ± 0.09	1.17 ± 0.42	2.04 ± 0.56
stomach	2.89 ± 1.53	1.54 ± 0.36	0.44 ± 0.31	1.17 ± 0.20
intestine	2.47 ± 0.53	3.30 ± 1.22	3.28 ± 1.20	3.26 ± 0.89
blood	7.17 ± 0.54	6.40 ± 0.38	7.46 ± 0.35	7.87 ± 0.28
spleen	4.81 ± 0.51	4.24 ± 0.12	3.82 ± 0.60	4.32 ± 0.36
tumor	7.20 ± 0.27	2.35 ± 0.14	2.57 ± 0.20	1.98 ± 0.13
skin	0.56 ± 0.01	0.33 ± 0.02	0.37 ± 0.03	0.37 ± 0.05
muscle	1.42 ± 0.20	1.18 ± 0.15	1.28 ± 0.03	1.39 ± 0.14

Values are mean ± SD and expressed as %ID/g (n = 30).

Supplementary Table 3: Biodistribution of ^{99m}Tc-HYNIC-MPG at 4 h time point

Organ	PC9	H1975	H358	H520
**brain**	0.02 ± 0.00	0.09 ± 0.00	0.08 ± 0.00	0.08 ± 0.01
**heart**	0.27 ± 0.02	0.27 ± 0.01	0.28 ± 0.00	0.28 ± 0.00
**lung**	2.70 ± 0.36	2.52 ± 0.33	3.61 ± 0.32	3.33 ± 0.11
**liver**	10.12 ± 1.76	8.16 ± 0.71	7.57 ± 0.82	7.71 ± 0.75
**bone**	0.65 ± 0.05	0.48 ± 0.02	0.45 ± 0.04	0.59 ± 0.02
**kidney**	1.45 ± 0.66	1.10 ± 0.50	1.57 ± 0.08	1.29 ± 0.65
**stomach**	1.58 ± 0.13	1.47 ± 0.04	1.49 ± 0.26	1.92 ± 0.08
**intestine**	3.31 ± 0.38	0.33 ± 0.08	3.34 ± 0.56	2.36 ± 1.03
**blood**	3.49 ± 0.07	3.50 ± 0.24	3.34 ± 0.10	3.68 ± 0.27
**spleen**	4.28 ± 0.15	3.50 ± 0.24	2.77 ± 0.42	2.37 ± 0.33
**tumor**	5.30 ± 0.28	1.91 ± 0.10	1.45 ± 0.25	1.44 ± 0.47
**skin**	0.26 ± 0.06	0.27 ± 0.01	0.25 ± 0.01	0.26 ± 0.00
**muscle**	1.36 ± 0.15	1.19 ± 0.16	1.36 ± 0.06	1.11 ± 0.05

Values are mean ± SD and expressed as %ID/g (n = 3).

Supplementary Table 4: Biodistribution of ^{99m}Tc-HYNIC-MPG at 6 h time point

Organ	PC9	H1975	H358	H520
**brain**	0.02 ± 0.00	0.03 ± 0.00	0.01 ± 0.00	0.02 ± 0.00
**heart**	0.20 ± 0.00	0.23 ± 0.02	0.21 ± 0.00	0.20 ± 0.01
**lung**	2.55 ± 0.25	2.26 ± 0.20	2.16 ± 0.16	2.49 ± 0.06
**liver**	5.07 ± 0.74	4.63 ± 0.10	6.34 ± 0.60	5.72 ± 0.70
**bone**	0.29 ± 0.01	0.24 ± 0.03	0.24 ± 0.01	0.26 ± 0.02
**kidney**	0.69 ± 0.23	0.83 ± 0.14	1.48 ± 0.11	1.34 ± 0.40
**stomach**	0.22 ± 0.02	1.57 ± 0.08	1.73 ± 0.13	1.60 ± 0.17
**intestine**	3.91 ± 0.96	0.24 ± 0.03	3.25 ± 0.76	2.25 ± 0.63
**blood**	1.59 ± 0.11	1.56 ± 0.07	1.60 ± 0.09	1.52 ± 0.02
**spleen**	1.52 ± 0.11	1.30 ± 0.03	1.25 ± 0.10	1.54 ± 0.11
**tumor**	3.03 ± 0.12	1.51 ± 0.24	0.67 ± 0.21	0.37 ± 0.05
**skin**	0.19 ± 0.01	0.14 ± 0.01	0.15 ± 0.01	0.13 ± 0.02
**muscle**	0.81 ± 0.08	0.81 ± 0.09	0.83 ± 0.12	0.84 ± 0.08

Values are mean ± SD and expressed as %ID/g (n = 3).
